# Synthesis and Antifungal Activity of *N*-(Substituted pyridinyl)-1-methyl(phenyl)-3-(trifluoromethyl)-1*H*-pyrazole-4-carboxamide Derivatives

**DOI:** 10.3390/molecules171214205

**Published:** 2012-11-30

**Authors:** Zhibing Wu, Deyu Hu, Jiqing Kuang, Hua Cai, Shixi Wu, Wei Xue

**Affiliations:** State Key Laboratory Breeding Base of Green Pesticides and Agricultural Bioengineering, Key Laboratory of Green Pesticides and Agricultural Bioengineering, Ministry of Education, Guizhou University, Guiyang 550025, China; E-Mails: wzb1171@163.com (Z.W.); fcc.dyhu@gzu.edu.cn (D.H.); kuangjiqing@yeah.net (J.K.); woshicaihua@126.com (H.C.); gzushixiwu@163.com (S.W.)

**Keywords:** pyrazolecarboxamide derivatives, synthesis, antifungal activity, fungi

## Abstract

A series of *N*-(substituted pyridinyl)-1-methyl(phenyl)-3-trifluoromethyl-1*H*-pyrazole-4-carboxamide derivatives were synthesized. All target compounds were characterized by spectral data (^1^H-NMR, ^13^C-NMR, IR, MS) and elemental analysis and were bioassayed *in vitro* against three kinds of phytopathogenic fungi (*Gibberella zeae*, *Fusarium oxysporum*, *Cytospora mandshurica*). The results showed that some of the synthesized *N*-(substituted pyridinyl)-1-methyl-3-trifluoromethyl-1*H*-pyrazole-4-carboxamides exhibited moderate antifungal activities, among which compounds **6a**, **6b** and **6c** displayed more than 50% inhibition activities against *G. zeae* at 100 µg/mL, which was better than that of the commercial fungicides carboxin and boscalid.

## 1. Introduction

The mitochondria are membrane enclosed organelles found in most eukaryotic cells [1], which are usually described as “cellular power plants” because they generate most of the cell’s supply of adenosine triphosphate (ATP), used as a source of chemical energy [2], and they also play an important role in plant protection against many phytopathogenic fungi. Carboxanilides are traditional active compounds which have been applied in agro-chemistry [3,4,5], and different kinds of commercial products based on the carboxanilide structure have been developed in recent years. Early carboxanilide fungicides such as carboxin have special activity against basidiomycetes, but limited activity towards other plant pathogens [6,7]. The mode of action for these commercial fungicides revealed that they can specifically bind to the ubiquinone-binding site (Q-site) of the mitochondrial complex II, interrupting electron transport in the mitochondrial respiratory chain, so the fungi can’t produce vital energy to form ATP [8,9,10,11].

**Figure 1:**
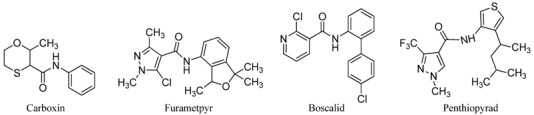
The commercial carboxamide fungicides.

The results of structure-activity relationships (SAR) research revealed that highly active novel carboxanilide derivatives containing two heterocyclic rings and alkyl substitution at the amino heterocyclic ring such as furametpyr and penthiopyrad (Figure 1) show an expanded antifungal spectrum [5,12]. Both of these commercial carboxamide fungicides have remarkable activity, not only against basidiomycetes, but also the ascomycetes (such as gray mold, powdery mildew, and apple scab) [7,12,13].

**Scheme 1:**
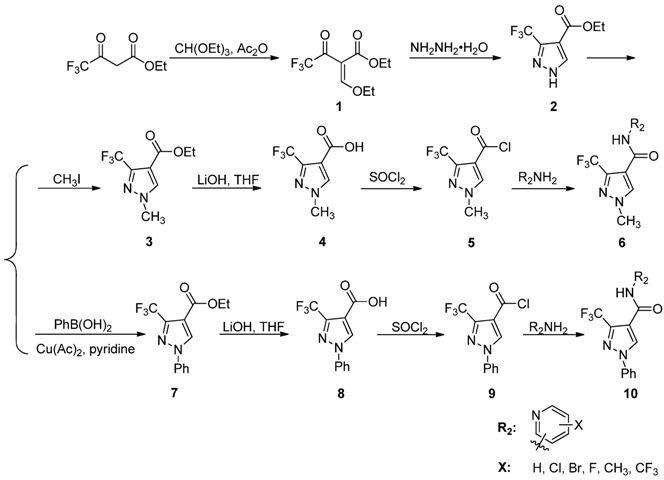
Synthetic route to compounds **6a**–**l** and **10a**–**k**.

Penthiopyrad is one novel carboxamide fungicide, whose mode of fungicidal action indicates that it inhibits succinate dehydrogenase and this results in the inhibition of the citric acid cycle and mitochondrial electron transport pathways. The pyridine moiety is a traditional group with broad activity which is often used in the molecular design of agrochemicals. Based on the molecular structure of penthiopyrad, a new series of pyrazole carboxamide compounds containing an active 3-trifluoromethyl-1*H*-pyrazole-4-carboxamide skeleton have been designed by introducing active pyridine rings into the amino part of pyrazole carboxamide. In this work 12 novel *N*-(substituted pyridinyl)-1-methyl-3-trifluoromethylpyrazole carboxamide analogues and 11 novel *N*-(substituted pyridinyl)-1-phenyl-3-trifluoromethylpyrazole carboxamide analogues were synthesized (Scheme 1). The antifungal activity against *Gibberella zeae* (*G. zeae*), *Fusarium oxysporium* (*F. oxysporium*), *Cytospora mandshurica* (*C. mandshurica*) were then evaluated.

## 2. Results and Discussion

### 2.1. Chemistry

The synthetic protocols of the target compounds are depicted in Scheme 1. Intermediate **2** was prepared by reacting compound **1** with hydrazine hydrate [14]. The intermediates **3** and **7** were synthesized from ethyl 3-(trifluoromethyl)-1*H*-pyrazole-4-carboxylate (**2**) with different methods. Ethyl 1-methyl-3-(trifluoromethyl)-1*H*-pyrazole-4-carboxylate (**3**) can be prepared via substitution reaction of **2** with iodomethane [15], and ethyl 1-phenyl-3-(trifluoromethyl)-1*H*-pyrazole-4-carboxylate (**7**) can be synthesized by a Suzuki coupling reaction of **2** with phenylboronic acid [16].

Then intermediates **3** and **7** reacted with lithium hydroxide in THF to afford pyrazole acids **4** and **8**, respectively. Subsequently **4** and **8** were refluxed in SOCl_2_ to give pyrazole acid chlorides **5** and **9**. Compounds **6a**–**l** and **10a**–**k** were then obtained by reaction of **5** or **9** with different substituted pyridylamines, respectively [17,18]. The ^1^H-NMR, ^13^C-NMR, IR and MS data and elemental analysis for the synthesized new compounds were consistent with assigned structures.

### 2.2. Antifungal Activity

As indicated at Table 1, most of the synthesized compounds possessed antifungal activity to a certain extent, and some of the synthesized compounds exhibited moderately antifungal activity, such as **6a**, **6b** and **6c** that displayed more than 50% inhibition activity against *G. zeae* at 100 µg/mL, which were better than that of the commercial fungicides carboxin and boscalid. The inhibition rates of compound **6a** against *G. zeae*, *F. oxysporum* and *C. mandshurica* were 73.2%, 53.5%, 48.7% respectively, better than that of the other compounds.

**Table 1:** Inhibition effect of compound **6a**–**l** and **10a**–**k** against phytopathogenic fungi at 100 µg/mL. 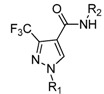

Compound	R_1_	R_2_	Inhibition rate ^a^ (%)		
			*G. zeae*	*F. oxysporum*	*C. mandshurica*
**6a**	CH_3_	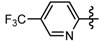	51.6 ± 1.3	39.1 ± 1.4	35.6 ± 2.2
**6b**	CH_3_	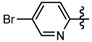	73.2 ± 1.7	53.5 ± 1.5	48.7 ± 2.0
**6c**	CH_3_	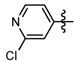	60.1 ± 1.0	5.9 ± 1.2	9.1 ± 1.6
**6d**	CH_3_	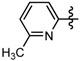	34.7 ± 0.9	22.27 ± 1.3	20.8 ± 1.5
**6e**	CH_3_	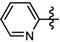	40.9 ± 1.7	19.5 ± 0.8	9.0 ± 1.9
**6f**	CH_3_	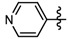	26.3 ± 1.0	11.8 ± 1.5	2.9 ± 1.7
**6g**	CH_3_	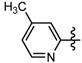	25.2 ± 8.2	6.9 ± 1.1	1.74 ± 1.6
**6h**	CH_3_	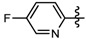	30.4 ± 1.8	22.5 ± 0.9	13.6 ± 1.5
**6i**	CH_3_	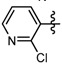	40.4 ± 1.1	14.9 ± 0.9	4.9 ± 2.2
**6j**	CH_3_	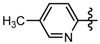	39.3 ± 1.5	8.3 ± 0.8	0
**6k**	CH_3_	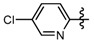	26.0 ± 1.4	30.1 ± 1.1	13.6 ± 1.8
**6l**	CH_3_	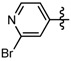	25.2 ± 1.8	10.9 ± 1.8	6.4 ± 1.3
**10a**	Ph	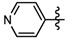	11.3 ± 1.0	7.9 ± 2.1	0
**10b**	Ph	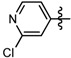	16.2 ± 1.9	0	0
**10c**	Ph	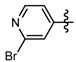	10.7 ± 1.2	5.7 ± 1.2	0
**10d**	Ph	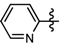	35.2 ± 1.6	23.9 ± 2.1	17.0 ± 1.2
**10f**	Ph	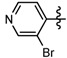	22.6 ± 1.3	27.2 ± 2.9	8.4 ± 1.0
**10g**	Ph	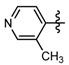	6.7 ± 1.1	11.5 ± 3.6	6.1 ± 0.8
**10h**	Ph	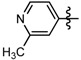	9.8 ± 0.9	4.23 ± 1.7	0
**10i**	Ph	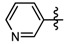	6.1 ± 1.2	10.6 ± 3.8	0
**10j**	Ph	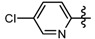	16.5 ± 1.2	14.2 ± 1.0	10.1 ± 0.9
**10k**	Ph	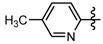	6.4 ± 1.5	20.5 ± 0.8	0
Carboxin			56.3 ± 2.4	23.6 ± 9.4	--
Boscalid			35.9 ± 3.5	4.3 ± 1.3	3.4 ± 1.5

^a^ Average of three replicates. “--” Not tested.

### 2.3. Toxicity of Target Compound ***6b*** on Three Kinds of Pathogenic Fungi

The EC_50_ values of compound **6b** against three plant pathogens (*G. zeae*, *F. oxysporum* and *C. mandshurica*) were 81.3 µg/mL, 97.8 µg/mL, 176.5 µg/mL, respectively (Table 2). The preliminary structure-activity relationship analysis indicated that the antifungal activity of the synthesized compounds was obviously decreased when the methyl group at N-1 of the pyrazole ring was replaced by a phenyl group.

**Table 2:** Toxicity of compound **6b** on three kinds of pathogenic fungi ^a^.

Compounds	Fungi	Toxic regression equation	EC_50_ (μg/mL)	R
**6b**	*G. zeae*	y = 2.8606x − 0.4639	76.3 ± 1.3	0.949
	*F. oxysporum*	y = 1.3314x + 2.2993	97.8 ± 2.7	0.983
	*C. mandshurica*	y = 0.7853x + 3.1312	132.5 ± 2.1	0.967

^a^ Average of three replicates.

## 3. Experimental

### 3.1. Chemistry

All the melting points were determined with a XT-4 binocular microscope melting point apparatus (Beijing Tech Instrument Co., Beijing, China), while the thermometer was uncorrected. ^1^H-NMR and ^13^C-NMR were recorded with a JEOL-ECX500 NMR spectrometer operating at 500 MHz and 125 MHz respectively, and tetramethylsilane (TMS) was used as the internal standard. Mass spectral studies were conducted on an Agilent 5973 organic mass spectrometer. Elemental analysis was performed on an Elementar Vario-III CHN analyzer (Hanau, Germany). Analytical thin-layer chromatography (TLC) was performed on silica gel GF254. Column chromatographic purification was carried out using silica gel, the spots were visualized with ultraviolet (UV) light. All reagents were of analytical reagent grade or chemically pure. All solvents were dried, deoxygenated, and redistilled before use.

### 3.2. Ethyl 3-(trifluoromethyl)-1H-pyrazole-4-carboxylate (***2***)

A mixture of ethyl 3,3,3-trifluoro-2-*oxo*-butanoate (30.0 g, 0.16 mol), triethyl orthoformate (75.7 g, 0.33 mol) and acetic anhydride (49.0 g, 0.49 mol) was stirred at 120 °C for 12 h. The reaction mixture was concentrated *in vacuo*. The residue was dissolved in methanol (150 mL), hydrazine hydrate (12.2 g, 0.24 mol) was added, and reacted at 80 °C for 3 h. The reaction mixture was concentrated *in vacuo*. The residue was extracted with ethyl acetate, washed with water and brine, dried over anhydrous magnesium sulfate and concentrated *in vacuo*. The residue was recrystallized from ethyl acetate-hexane to give compound **2** as a light yellow solid (20.4 g, yield 60.0%), m.p. 141~142 °C. ^1^H-NMR (DMSO-*d_6_*): 14.13 (s, 1H, pyrazole NH), 8.59 (s, 1H, pyrazole H), 4.24 (q, *J* = 7.2 Hz, 2H, CH_2_), 1.27 (t, *J* = 7.2 Hz, 3H, CH_3_).

### 3.3. Ethyl 1-methyl-3-(trifluoromethyl)-1H-pyrazole-4-carboxylate (***3***)

Ethyl 3-(trifluoromethyl)-1*H*-pyrazole-4-carboxylate (**2**, 5.0 g, 21.2 mmol) and NaH (1.3 g, 42.4 mmol) were dissolved in DMF (30 mL), CH_3_I (3.3 g, 23.3 mmol) in DMF (10 mL) was added slowly into the solution while the temperature was kept under 0 °C, and reacted for 20 min, then the resulting mixture was monitored by TLC and reacted at ambient temperature for about 20 h. After completion of the reaction, water was added, and the reaction mixture was subsequently extracted with ethyl acetate, the organic layer were washed with brine, dried over anhydrous magnesium sulfate and concentrated *in vacuo*. The residue was recrystallized from ethyl acetate-petroleum to give compound **3** as a white crystalline solid (8.2 g, yield 73.8%), m.p. 58~60 °C; ^1^H-NMR (DMSO-*d_6_*) δ: 8.55(s, 1H, pyrazole H), 4.24 (q, *J* = 7.2 Hz, 2H, OCH_2_CH_3_), 3.95 (s, 3H, *N*-CH_3_), 1.27 (t, *J* = 7.2 Hz, 3H, OCH_2_CH_3_); MS (ESI) *m/z*: 223 [M+H]^+^.

### 3.4. Ethyl 1-Phenyl-3-(trifluoromethyl)-1H-pyrazole-4-carboxylate (***7***)

Ethyl 3-(trifluoromethyl)-1*H*-pyrazole-4-carboxylate (**2**, 8.0 g, 38.4 mmol) was dissolved in DMF (80 mL), then phenylboronic acid (9.4 g, 76.8 mmol), cupric acetate (10.5 g, 57.7 mmol) and pyridine (6.1 g, 76.9 mmol) were added to the solution, the resulting mixture was monitored by TLC and reacted at ambient temperature for about 24 h, then filtrated with kieselguhr, and extracted with ethyl acetate, the organic layer were washed with NaHCO_3_, dried over anhydrous magnesium sulfate and concentrated *in vacuo*. The residue was recrystallized from ethanol to give compound **7** as a white crystalline solid, yield 91.0%, m.p. 89~90 °C; ^1^H-NMR (DMSO-*d_6_*): 9.33 (s, 1H, pyrazole H), 7.95 (d, *J* = 8.6 Hz, 2H, benzene H), 7.58 (t, *J* = 8.0 Hz, 2H, benzene H), 7.48 (t, *J* = 7.5 Hz, 1H, benzene H), 4.31 (q, *J* = 7.2 Hz, 2H, CH_2_), 1.32 (t, *J* = 7.2 Hz, 3H, CH_3_); MS (ESI): *m/z* 285 [M+H]^+^.

### 3.5. General Synthetic Procedure for Intermediates ***4*** and ***8***

To a solution of compound **3** (4.0 g, 18.0 mmol) in THF (20 mL), lithium hydroxide (1.72 g, 0.72 mol) were added, reacted at 70 °C for about 2 h. The solution was concentrated *in vacuo*, the pH was adjusted to 5 with hydrochloric acid, the reaction mixture was filtered, and the filtrate was washed with water to give 1-methyl-3-(trifluoromethyl)-1*H*-pyrazole-4-carboxylic acid (**4**), as a white crystalline solid (3.1 g, yield 89.2%), m.p. 133–134 °C; ^1^H-NMR (DMSO-*d_6_*) δ: 12.90 (s, 1H, COOH), 8.46 (s, 1H, pyrazole H), 3.93 (s, 3H, *N-*CH_3_); MS (ESI) *m/z*: 195 [M+H]^+^.

Intermediate **8** (1-phenyl-3-(trifluoromethyl)-1*H*-pyrazole-4-carboxylic acid) can be prepared by the same method as **4**. White solid, yield 97.6%, m.p. 223~224 °C; ^1^H-NMR (DMSO-*d_6_*): 9.23 (s, 1H, pyrazole H), 7.93 (d, *J* = 8.6 Hz, 2H, benzene H), 7.57 (t, *J* = 7.8 Hz, 2H, benzene H), 7.47 (t, *J* = 7.2 Hz, 1H, benzene H); MS (ESI): *m/z* 257 [M+H]^+^.

### 3.6. General Synthetic Procedure for Intermediates ***5*** and ***9***

1-Methyl-3-(trifluoromethyl)-1*H*-pyrazole-4-carbonyl chloride (**5**) and 1-phenyl-3-(trifluoromethyl)-1*H*-pyrazole-4-carbonyl chloride (**9**) were prepared by refluxing **4** and **8** in thionyl chloride for 8 h, respectively.

### 3.7. Preparation of the Target Compounds ***6*** and ***10***

To a solution of 2-amino-5-(trifluoromethyl) pyridine (163 mg, 1 mmol) and 80% sodium hydride (45 mg, 1.5 mmol) in anhydrous THF (10 mL), compound **5** (214 mg, 1 mmol) was added slowly, while the temperature was controlled under 10 °C, then reacted at ambient temperature for 10 h. The solvent was distilled off, and the residue was dissolved in ethyl acetate, washed with water and brine, dried over by anhydrous sodium sulfate. After the solvent was removed under reduced pressure, the residue was purified by column chromatography on silica gel (ethyl acetate/petroleum ether 1:15) to afford the target product, *N*-(5-(trifluoromethyl)-pyridin-2-yl)-1-methyl-3-(trifluoro-methyl)-1*H*-pyrazole-4-carboxamide (**6a**). The following compounds **6b**–**l** and **10a**–**k** was prepared from the corresponding starting materials in a similar manner to that described for **6a**. The physical and spectral data for compounds **6b**–**l** and **10a**–**k** are listed below.

*1-Methyl-3-(trifluoromethyl)-N-(5-(trifluoromethyl)pyridin-2-yl)-1H-pyrazole-4-carboxamide* (**6a**): White solid, yield 86.4%, m.p. 128~130 °C. ^1^H-NMR (DMSO-*d_6_*) δ: 11.19 (s, 1H, NH), 8.76 (s, 1H, pyrazole H), 8.73 (1H, pyridine H), 8.34 (d, *J* = 8.6 Hz, 1H, pyridine H), 8.20 (d, *J* = 8.6 Hz, 1H, pyridine H), 3.97 (s, 3H, *N*-CH_3_); ^13^C-NMR (DMSO-*d_6_*): δ 160.03, 155.60, 145.82, 136.42, 136.07, 123.3, 122.40, 121.25, 120.27, 115.68, 114.21, 40.51; IR (KBr): *ν* 3305.9, 3121.8, 1681.9, 1591.1, 1542.3, 1525.6, 1051.2, 848.6, 752.5 cm^−1^; MS(ESI): *m/z* 339 [M+H]^+^; Anal. Calc. for C_12_H_8_F_6_N_4_O: C, 42.62; H, 2.38; N, 16.57. Found: C, 42.22; H, 2.05; N, 16.11.

*1-Methyl-N-(5-bromopyridin-2-yl)-3-(trifluoromethyl)-1H-pyrazole-4-carboxamide* (**6b**): White solid, yield 74.2%, m.p. 170~173 °C. ^1^H-NMR (DMSO-*d_6_*) δ: 10.91 (s, 1H, NH), 8.68 (s, 1H, pyrazole H), 8.49 (s, 1H, pyridine H), 8.12 (d, *J* = 9.15 Hz, 1H, pyridine H), 8.03 (d, *J* = 8.6 Hz, 1H, pyridine H), 3.94 (s, 3H, *N*-CH_3_); ^13^C-NMR (DMSO-*d_6_*): δ 159.67, 151.45, 149.03, 141.18, 135.81, 122.44, 120.30, 116.39, 115.94, 114.38, 40.48; IR (KBr): *ν* 3429.4, 3128.5, 3261.6, 1674.2, 1543.0, 1521.1, 1496.7, 1053.1, 835.1 cm^−1^; MS(ESI) *m/z*: 349 [M+H]^+^; Anal. Calc. for C_11_H_8_BrF_3_N_4_O: C, 37.84; H, 2.31; N, 16.05. Found: C, 37.38; H, 1.99; N, 15.75.

*1-Methyl-N-(2-chloropyridin-4-yl)-3-(trifluoromethyl)-1H-pyrazole-4-carboxamide* (**6c**): White solid, yield 45.1%, m.p. 129~131 °C. ^1^H-NMR (DMSO-*d_6_*) δ:10.00 (s, 1H, NH), 8.59 (s, 1H, pyrazole H), 8.29 (1H, pyridine H), 8.07 (s, 1H, pyridine H), 7.49 (1H, pyridine H), 3.97 (s, 3H, N-CH_3_); ^13^C-NMR (DMSO-*d_6_*): δ 159.43, 147.06, 145.98, 136.80, 135.43, 132.02, 124.01, 122.40, 120.27, 115.79, 40.51; IR (KBr): *ν* 3284.7, 3121.1, 1660.7, 1657.7, 1548.8, 1541.1, 1521.8, 1456.2, 1496.7, 1078.21, 1051.1, 800 cm^−1^; MS(ESI): *m/z* 305 [M+H]^+^; Anal. Calc. for C_11_H_8_ClF_3_N_4_O: C, 43.37; H, 2.65; N, 18.39. Found: C, 43.11; H, 2.34; N, 17.93.

*1-Methyl-N-(6-methylpyridin-2-yl)-3-(trifluoromethyl)-1H-pyrazole-4-carboxamide* (**6d**): Light yellow solid, yield 66.3%, m.p. 133~136 °C. ^1^H-NMR (DMSO-*d_6_*) δ: 10.61(s, 1H, NH), 8.66 (s, 1H, pyrazole H), 8.19 (m, 1H, pyridine H), 8.02 (d, *J* = 8.0 Hz, 1H, pyridine H), 7.63 (d, *J* = 8.0 Hz, 1H, pyridine H), 3.96 (s, 3H, N-CH_3_); 2.26 (s, 3H, CH_3_); ^13^C-NMR (DMSO-*d_6_*): δ 159.41, 150.27, 148.19, 139.05, 135.52, 129.21, 122.51, 120.36, 116.33, 114.32, 40.49, 17.80; IR (KBr): *ν* 3365.7, 3130.4, 1678.0, 1591.2, 1541.1, 1533.4, 1330.8, 1282.6, 869.9 cm^−1^; MS(ESI): *m/z* 285 [M+H]^+^; Anal. Calc. for C_12_H_11_F_3_N_4_O: C, 50.71; H, 3.90; N, 19.71. Found: C, 50.53; H, 3.65; N, 19.48.

*1-Methyl-N-(pyridin-2-yl)-3-(trifluoromethyl)-1H-pyrazole-4-carboxamide* (**6e**): White solid, yield 75.6%, m.p. 159~162 °C. ^1^H-NMR (DMSO-*d_6_*) δ: 10.70 (s, 1H, NH), 8.68 (s, H, pyrazole H), 8.36 (d, *J* = 3.5 Hz, 1H, pyridine H), 8.13 (d, *J* = 8.6 Hz, 1H, pyridine H), 7.81 (t, *J* = 7.7 Hz, 1H, pyridine H), 7.15 (t, *J* = 6.3 Hz, 1H, pyridine H), 3.96 (s, 3H, CH_3_); ^13^C-NMR (DMSO-*d_6_*): δ 159.62, 152.46, 148.47, 140.13, 138.73, 135.65, 120.29, 116.25, 115.81, 114.78, 40.54; IR(KBr): *ν* 3312.1, 3226.9, 1683.8, 1579.7, 1541.1, 1527.6, 1506.4, 1055.0, 823.6, 790.8, 754.1 cm^−1^; MS(ESI): *m/z* 271 [M+H]^+^; Anal. Calc. for C_11_H_9_F_3_N_4_O: C, 48.89; H, 3.36; N, 20.73. Found: C, 48.44; H, 3.08; N, 20.22.

*1-Methyl-N-(pyridin-4-yl)-3-(trifluoromethyl)-1H-pyrazole-4-carboxamide* (**6f**): White solid, yield 59.7%, m.p. 205~208 °C. ^1^H-NMR (DMSO-*d_6_*) δ:10.46 (s, 1H, NH), 8.57(s, H, pyrazole H), 8.46 (d, *J* = 1.7 Hz, 1H, pyridine H), 8.45 (d, *J* = 1.2 Hz, 1H, pyridine H), 7.66 (d, *J* = 1.2 Hz, 1H, pyridine H), 7.65 (d, *J* = 1.7 Hz, 1H, pyridine H), 3.99 (s, 3H, CH_3_); ^13^C-NMR (DMSO-*d_6_*): δ 159.94, 150.93, 146.11, 139.96, 135.37, 122.39, 116.31, 114.12, 40.53; IR (KBr): *ν* 3044.1, 3226.9, 1697.3, 1583.5, 1570.0, 1541.1, 1003.8, 835.1, 775.3 cm^−1^; MS(ESI): *m/z* 271 [M+H]^+^; Anal. Calc. for C_11_H_9_F_3_N_4_O: C, 48.89; H, 3.36; N, 20.73. Found: C, 48.56; H, 3.01; N, 20.39.

*1-Methyl-N-(4-methylpyridin-2-yl)-3-(trifluoromethyl)-1H-pyrazole-4-carboxamide* (**6g**): Light yellow solid, yield 47.8%, m.p. 138~141 °C, ^1^H-NMR (DMSO-*d_6_*) δ: 10.61 (s, 1H, NH), 8.41 (s, 1H, pyrazole H), 8.19 (s, 1H, pyridine H), 7.71 (d, *J* = 5.2 Hz, 1H, pyridine H), 7.17 (d, *J* = 5.2 Hz, 1H, pyridine H), 3.89 (s, 3H, N-CH_3_); 2.30 (s, 3H, CH_3_); ^13^C-NMR (DMSO-*d_6_*): δ 160.04, 152.82, 150.68, 140.73, 136.64, 124.80, 119.95, 116.56, 115.15, 113.91, 40.49, 21.12; IR (KBr): *ν* 3363.8, 3112.5, 1670.3, 1575.8, 1541.1, 1506.4, 1456.2, 1055.0, 873.7, 825.5, 752.4 cm^−1^; MS(ESI): *m/z* 285 [M+H]^+^; Anal. Calc. for C_12_H_11_F_3_N_4_O: C, 50.71; H, 3.90; N, 19.71. Found: C, 50.27; H, 3.66; N, 19.54.

*1-Methyl-N-(5-fluoropyridin-2-yl)-3-(trifluoromethyl)-1H-pyrazole-4-carboxamide* (**6h**): Yellow solid, yield 82.4%, m.p. 135~137 °C. ^1^H-NMR (DMSO-*d_6_*) δ: 10.83 (s, 1H, NH), 8.65 (s, H, pyrazole H), 8.37(d, 1H, pyridine H), 8.16 (1H, pyridine H), 7.76 (1H, pyridine H), 3.96 (s, 3H, CH_3_); ^13^C-NMR (DMSO-*d_6_*): δ 160.07, 151.35, 147.66, 140.10, 137.88, 125.26, 120.23, 119.17, 118.52, 114.73, 40.50; IR (KBr): *ν* 3.454.5, 3136.2, 1670.3, 1541.1, 1521.8, 1489.0, 1392.6, 1055.0, 840.1, 771.2 cm^−1^; MS(ESI): *m/z* 289 [M+H]^+^; Anal. Calc. for C_11_H_8_F_4_N_4_O: C, 45.84; H, 2.80; N, 19.44. Found: C, 45.56; H, 2.63; N, 19.14.

*1-Methyl-N-(2-chloropyridin-3-yl)-3-(trifluoromethyl)-1H-pyrazole-4-carboxamide* (**6i**): Light yellow solid, yield 33.3%, m.p. 107~109 °C. ^1^H-NMR (DMSO-*d_6_*) δ: 10.25 (s, 1H, NH), 8.48 (s, 1H, pyrazole H), 8.34 (d, *J* = 7.5 Hz,1H, pyridine H), 8.03 (d, *J* = 8.0 Hz, 1H, pyridine ), 7.59 (q, *J* = 4.0 Hz, 1H, pyridine H), 3.89 (s, 3H, CH_3_); ^13^C-NMR (DMSO-*d_6_*): δ 163.57, 150.55, 149.23, 140.76, 136.72, 133.98, 125.19, 121.98, 119.84, 115.68, 40.51; IR (KBr): *ν* 3415.5, 3127.1, 1691.5, 1585.4, 1541.1, 1514.1, 1490.9, 1037.7, 815.8, 763.8, 732.9 cm^−1^; MS(ESI): *m/z* 305 [M+H]^+^; Anal. Calc. for C_11_H_8_ClF_3_N_4_O: C, 43.37; H, 2.65; N, 18.39. Found: C, 42.88; H, 2.14; N, 18.25.

*1-Methyl-N-(5-methylpyridin-2-yl)-3-(trifluoromethyl)-1H-pyrazole-4-carboxamide* (**6j**): Yellow solid, yield 43.9%, m.p. 135~138 °C. ^1^H-NMR (DMSO-*d_6_*) δ: 10.34 (s, 1H, NH), 8.80 (s, 1H, pyrazole H), 8.53 (s, 1H, pyridine H), 8.29 (d, *J* = 7.2 Hz, 1H, pyridine H), 8.11 (d, *J* = 7.2 Hz, 1H, pyridine H), 3.99 (s, 3H, N-CH_3_); 2.26 (s, 3H, CH_3_); ^13^C-NMR (DMSO-*d_6_*): δ 159.55, 151.23, 145.11, 141.89, 139.82, 135.11, 127.36, 124.18, 116.48, 115.94, 40.50, 24.37; IR (KBr): *ν* 3545.1, 3105.3, 1653.0, 1589.3, 1506.4, 1496.7, 1473.6, 1039.6, 891.1, 808.7, 789.9. cm^−1^; MS(ESI): *m/z* 285 [M+H]^+^; Anal. Calc. for C_12_H_11_F_3_N_4_O: C, 50.71; H, 3.90; N, 19.71. Found: C, 50.43; H, 3.77; N, 19.62.

*1-Methyl-N-(5-chloropyridin-2-yl)-3-(trifluoromethyl)-1H-pyrazole-4-carboxamide* (**6k**): White solid, yield 30.1%, m.p. 138~140 °C, ^1^H-NMR (DMSO-*d_6_*) δ: 10.14 (s, 1H, NH), 8.41 (s, 1H, pyrazole H), 8.15 (d, *J* = 9.2 Hz, 1H, pyridine H), 8.10 (s, 1H, pyridine H), 7.94 (d, *J* = 7.5 Hz, 1H, pyridine H), 4.03 (s, 3H, NCH_3_); ^13^C-NMR (DMSO-*d_6_*): δ 160.28, 150.99, 146.92, 139.30, 138.52, 126.18, 121.25, 119.95, 119.10, 115.84, 40.52; IR (KBr): *ν* 3282.8, 2964.5, 1701.2, 1575.8, 1525.6, 1456.2, 1292.8, 1155.3, 1006.8, 837.1, 736.8 cm^−1^; MS(ESI): *m/z* 305 [M+H]^+^; Anal. Calc. for C_11_H_8_ClF_3_N_4_O: C, 43.37; H, 2.65; N, 18.39. Found: C, 43.02; H, 2.18; N, 18.12.

*1-Methyl-N-(2-bromopyridin-4-yl)-3-(trifluoromethyl)-1H-pyrazole-4-carboxamide* (**6l**): White solid, yield 56.4%, m.p. 174~176 °C. ^1^H-NMR (DMSO-*d_6_*) δ: 10.67 (s, 1H, NH), 8.57 (s, 1H, pyrazole H), 8.29 (d, *J* = 5.8 Hz, 1H, pyridine H), 7.84 (s, 1H, pyridine H), 7.59 (d, *J* = 5.2 Hz, 1H, pyridine H), 3.99 (s, 3H, CH_3_); ^13^C-NMR (DMSO-*d_6_*): δ 160.05, 151.48, 150.95, 148.69, 135.55, 122.32, 120.17, 115.85, 113.53, 113.40, 40.50; IR (KBr): *ν* 3330.5, 3115.0, 1699.2, 1587.4, 1541.1, 1489.0, 1049.2, 842.1, 772.1 cm^−1^; MS(ESI): *m/z* 349 [M+H]^+^; Anal. Calc. for C_11_H_8_BrF_3_N_4_O: C, 37.84; H, 2.31; N, 16.05. Found: C, 37.39; H, 2.11; N, 15.87.

*N-(Pyridin-4-yl)-1-phenyl-3-(trifluoromethyl)-1H-pyrazole-4-carboxamide* **(10a)**: White solid, yield 80.5%, m.p. 244~245 °C. ^1^H-NMR (DMSO-*d_6_*) δ: 10.63 (s, 1H, NH), 9.35 (s, 1H, pyrazole H), 8.50 (d, *J* = 6.3 Hz, 2H, pyridine H), 7.89 (d, *J* = 8.1 Hz, 2H, benzene H), 7.69 (d, *J* = 6.3 Hz, 2H, pyridine H), 7.64 (t, *J* = 8.1 Hz, 2H, benzene H), 7.51 (t, *J* = 7.5 Hz, 1H, benzene H); ^13^C-NMR (DMSO-*d_6_*): δ 159.72, 151.04, 145.94, 141.81, 138.76, 136.44, 132.74, 130.54, 129.04, 120.10, 117.99, 114.14; IR (KBr): *ν* 3419.7, 3290.5, 2978.0, 1716.6, 1683.8, 1624.0, 1543.0, 1508.3, 1400.2, 1284.5 cm^−1^; MS(ESI): *m/z* 333 [M+H]^+^; Anal. Calc. for C_16_H_11_F_3_N_4_O: C, 57.83; H, 3.34; N, 16.86. Found: C, 57.65; H, 3.12; N, 16.54.

*N-(2-Chloropyridin-4-yl)-1-phenyl-3-(trifluoromethyl)-1H-pyrazole-4-carboxamide* (**10b**): White solid, yield 16.5%, m.p. 216~218 °C. ^1^H-NMR (DMSO-*d_6_*) δ: 10.23 (s, 1H, NH), 8.69 (s, 1H, pyrazole H), 8.51 (d, *J* = 5.8 Hz, 1H, pyridine H), 7.76 (d, *J* = 5.8 Hz, 1H, pyridine H), 7.58 (d, *J* = 8.1 Hz, 2H, benzene H), 7.43 (t, *J* = 7.6 Hz, 2H, benzene H), 7.51 (t, *J* = 7.5 Hz, 1H, benzene H), 7.36 (s, 1H, pyridine H); ^13^C-NMR (DMSO-*d_6_*): δ 159.35, 159.21, 149.78, 145.69, 138.66, 132.23, 129.97, 129.12, 125.54, 122.14, 120.04, 116.75, 114.03, 110.28; IR (KBr): *ν* 3444.8, 3383.1, 1701.2, 1653.0, 1577.7, 1490.9, 1356.8, 1298.0, 765.7 cm^−1^; MS(ESI): *m/z* 367 [M+H]^+^; Anal. Calc. for C_16_H_10_ClF_3_N_4_O: C, 52.40; H, 2.75; N, 15.28. Found: C, 52.22; H, 2.59; N, 15.04.

*N-(2-Bromopyridin-4-yl)-1-phenyl-3-(trifluoromethyl)-1H-pyrazole-4-carboxamide* (**10c**): White solid, yield 26.9%, m.p. 235~236 °C. ^1^H-NMR (DMSO-*d_6_*) δ: 10.83 (s, 1H, NH), 9.35 (s, 1H, pyrazole H), 8.35 (d, *J* = 5.7 Hz, 1H, pyridine H), 7.89 (s, *1*H, pyridine H), 7.87 (d, *J* = 3.4 Hz, 2H, benzene H), 7.65–7,61 (m, *3*H, benzene 2H and pyridine H), 7.51 (t, *J* = 7.2 Hz, 1H, benzene H); ^13^C-NMR (DMSO-*d_6_*): δ 159.86, 151.57, 151.12, 148.51, 143.56, 138.70, 132.98, 130.58, 129.15, 120.15, 117.56, 116.72, 113.56, 113.44; IR (KBr): *ν* 3365.8, 3111.1, 3066.8, 2922.1, 2850.7, 1705.0, 1579.7, 1543.0, 1494.8, 1386.8, 1298.0, 1043.4, 688.5 cm^−1^; MS(ESI): *m/z*411 [M+H]^+^, 412 [M+2H]^+^; Anal. Calc. for C_16_H_10_BrF_3_N_4_O: C, 46.74; H, 2.45; N, 13.63. Found: C, 46.34; H, 2.18; N, 13.47.

*N-(Pyridin-2-yl)-1-phenyl-3-(trifluoromethyl)-1H-pyrazole-4-carboxamide* (**10d**): White solid, yield 40.5%, m.p. 149~151 °C. ^1^H-NMR (DMSO-*d_6_*) δ: 10.85 (s, 1H, NH), 9.52 (s, 1H, pyrazole H), 8.23 (d, *J* = 8.0 Hz, 1H, benzene H), 7.87–7.49 (m, 9 H, pyridine and benzene H); ^13^C-NMR (DMSO-*d_6_*): δ 159.33, 152.36, 148.50, 138.81, 133.44, 132.97, 130.39, 128.82, 120.39, 120.35, 119.96, 118.36, 117.75, 114.64; IR (KBr): *ν* 3130.4, 3068.7, 2987.7, 2927.7, 1695.4, 1600.9, 1490.9, 1435.0, 1307.7 cm^−1^; MS(ESI): *m/z* 333 [M+H]^+^; Anal. Calc. for C_16_H_11_F_3_N_4_O: C, 57.83; H, 3.34; N, 16.86. Found: C, 57.66; H, 3.04; N, 16.49.

*N-(3-Chloropyridin-4-yl)-1-phenyl-3-(trifluoromethyl)-1H-pyrazole-4-carboxamide* (**10e**): White solid, yield 74.9%, m.p. 146~147 °C. ^1^H-NMR (DMSO-*d_6_*) δ: 10.08 (s, 1H, NH), 9.41 (s, 1H, pyridine H), 8.69 (s, 1H, pyrazole H), 8.51 (d, *J* = 5.7 Hz, 1H, pyridine H), 7.99 (d, *J* = 5.7 Hz, 1H, pyridine H), 7.91 (d, *J* = 8.0 Hz, 2H, benzene H), 7.64 (t, *J* = 7.8 Hz, 2H, benzene H), 7.51 (t, *J* = 7.5 Hz, 1H, benzene H); ^13^C-NMR (DMSO-*d_6_*): δ 159.46, 150.34, 149.26, 142.23, 138.76, 133.26, 130.49, 129.04, 123.34, 120.22, 119.04, 118.56, 117.31, 110.23; IR (KBr): *ν* 3387.0, 3101.5, 3034.0, 1707.0, 1581.6, 1496.7, 1406.1, 1294.2, 763.8 cm^−1^; MS(ESI): *m/z* 367 [M+H]^+^; Anal. Calc. for C_16_H_10_ClF_3_N_4_O: C, 52.40; H, 2.75; N, 15.28. Found: C, 51.98; H, 2.54; N, 14.96.

*N-(3-Bromopyridin-4-yl)-1-phenyl-3-(trifluoromethyl)-1H-pyrazole-4-carboxamide* (**10f**): White solid, yield 69.7%, m.p. 136~138 °C. ^1^H-NMR (DMSO-*d_6_*) δ: 9.98 (s, 1H, NH), 9.39 (s, 1H, pyridine H), 8.80 (s, 1H, pyrazole H), 8.53 (d, *J* = 5.2 Hz, 1H, pyridine H), 7.90–7.87 (m, *3*H, pyridine and benzene H), 7.63 (d, *J* = 8.0 Hz, 2H, benzene H), 7.64 (t, *J* = 7.8 Hz, 2H, benzene H), 7.51 (t, *J* = 6.9 Hz, 1H, benzene H); ^13^C-NMR (DMSO-*d_6_*): δ 159.26, 152.92, 149.69, 143.61, 138.76, 133.09, 130.49, 129.05, 122.21, 120.24, 120.07, 117.37, 114.93, 110.58; IR (KBr): *ν* 3400.5, 3325.2, 2924.0, 1670.3, 1579.7, 1494.8, 1406.8, 1235.3, 682.8 cm^−1^; MS(ESI): *m/z* 411 [M+H]^+^; Anal. Calc. for C_16_H_10_BrF_3_N_4_O: C, 46.74; H, 2.45; N, 13.63. Found: C, 46.57; H, 2.33; N, 13.47.

*N-(3-Methylpyridin-4-yl)-1-phenyl-3-(trifluoromethyl)-1H-pyrazole-4-carboxamide* (**10g**): White solid, yield 65.3%, m.p. 207~208 °C. ^1^H-NMR (DMSO-*d_6_*) δ: 9.83 (s, 1H, NH), 9.35 (s, 1H, pyridine H), 8. 44 (s, 1H, pyrazole H), 8.38 (d, *J* = 5.2 Hz, 1H, pyridine H), 7.90 (d, *J* = 8.0 Hz, 2H, benzene H), 7.73 (d, *J* = 5.2 Hz, 1H, benzene H), 7.63 (t, *J* = 6.9 Hz, 2H, benzene H), 7.51 (d, *J* = 6.3 Hz, 1H, pyridine H), 2.31 (s, 3H, CH_3_); ^13^C-NMR (DMSO-*d_6_*): δ 159.39, 152.05, 148.40, 143.70, 138.83, 132.86, 130.48, 128.97, 125.91, 120.16, 118.07, 117.78, 114.93, 111.23, 15.33; IR (KBr): *ν* 3244.2, 3116.9, 3030.1, 1699.2, 1581.6, 1544.9, 1490.9, 1409.9, 1300.0 cm^−1^; MS(ESI): *m/z*347 [M+H]^+^; Anal. Calc. for C_17_H_13_F_3_N_4_O: C, 58.96; H, 3.78; N, 16.18. Found: C, 58.66; H, 3.59; N, 16.01.

*N-(2-Methylpyridin-4-yl)-1-phenyl-3-(trifluoromethyl)-1H-pyrazole-4-carboxamide* (**10h**): White solid, yield 60.9%, m.p. 196~197 °C. ^1^H-NMR (DMSO-*d_6_*) δ: 10.53 (s, 1H, NH), 9.34 (s, 1H, pyridine H), 8.37 (s, 1H, pyrazole H), 7.88 (d, *J* = 8.0 Hz, 1H, pyridine H), 7.64–7.62 (m, *3*H, pyridine and benzene H), 7.50 (d, *J* = 9.0 Hz, 2H, benzene H), 2.46 (s, 3H, CH_3_); ^13^C-NMR (DMSO-*d_6_*): δ 159.64, 159.33, 150.27, 146.22, 138.76, 132.67, 130.54, 129.03, 126.12, 122.34, 120.10, 118.04, 113.03, 111.58, 24.84; IR (KBr): *ν* 3261.6, 3115.0, 3066.8, 1683.8, 1604.7, 1588.4, 1490.9, 1409.9, 1301.9 cm^−1^; MS(ESI): *m/z* 347 [M+H]^+^; Anal. Calc. for C_17_H_13_F_3_N_4_O: C, 58.96; H, 3.78; N, 16.18. Found: C, 58.67; H, 3.63; N, 15.94.

*N-(Pyridin-3-yl)-1-phenyl-3-(trifluoromethyl)-1H-pyrazole-4-carboxamide* (**10i**): White solid, yield 79.8%, m.p. 196~197 °C. ^1^H-NMR (DMSO-*d_6_*) δ: 10.50 (s, 1H, NH), 9.32 (s, 1H, pyridine H), 8.84 (s, 1H, pyrazole H), 8.33 (d, *J* = 3.5 Hz, 1H, pyridine H), 8.14 (d, *J* = 8.6 Hz, 1H, pyridine H), 7.88 (d, *J* = 7.5 Hz, 2H, benzene H), 7.63 (t, *J* = 8.0 Hz, 2H, benzene H), 7.50 (t, *J* = 7.5 Hz, 1H, benzene H), 7.42 q, *J* = 7.5 Hz, 1H, pyridine H); ^13^C-NMR (DMSO-*d_6_*): δ 161.11, 159.32, 145.34, 141.85, 138.79, 135.92, 132.50, 130.55, 129.00, 127.34, 124.30, 122.28, 120.07, 118.18; IR (KBr): *ν* 3259.7, 3122.7, 3066.8, 1695.4, 1653.0, 1600.9, 1490.9, 1419.6, 1303.8 cm^−1^; MS(ESI): *m/z* 333 [M+H]^+^; Anal. Calc. for C_16_H_11_F_3_N_4_O: C, 57.83; H, 3.34; N, 16.86. Found: C, 57.75; H, 3.16; N, 16.62.

*N-(5-Chloropyridin-2-yl)-1-phenyl-3-(trifluoromethyl)-1H-pyrazole-4-carboxamide* (**10j**): White solid, yield 67.8%, m.p. 101~102 °C. ^1^H-NMR (DMSO-*d_6_*) δ: 10.17 (s, 1H, NH), 9.36 (s, 1H, pyrazole H), 8.33 (d, *J* = 4.6 Hz, 1H, pyridine H), 8.13 (d, *J* = 8.1 Hz, 1H, pyridine H), 7.90 (d, *J* = 8.1 Hz, 2H, benzene H), 7.64 (t, *J* = 7.7 Hz, 2H, benzene H), 7.54–7.50 (m, 2H, benzene and pyrazole H); ^13^C-NMR (DMSO-*d_6_*): δ 160.97, 159.36, 146.94, 138.57, 135.39, 133.14, 130.49, 130.28, 129.00, 126.14, 120.30, 117.44, 115.71, 113.84; IR (KBr): *ν* 3421.7, 2926.0, 1705.0, 1653.0, 1575.8, 1506.4, 1386.8, 1298.0, 765.7 cm^−1^; MS(ESI): *m/z* 367 [M+H]^+^; Anal. Calc. for C_16_H_10_ClF_3_N_4_O: C, 52.40; H, 2.75; N, 15.28. Found: C, 52.03; H, 2.61; N, 14.98.

*N-(5-Methylpyridin-2-yl)-1-phenyl-3-(trifluoromethyl)-1H-pyrazole-4-carboxamide* (**10k**): White solid, yield 64.4%, m.p. 182~183 °C. ^1^H-NMR (DMSO-*d_6_*) δ: 11.17 (s, 1H, NH), 9.36 (s, 1H, pyrazole H), 8.33 (d, *J* = 4.6 Hz, 1H, pyridine H), 8.14 (d, *J* = 8.1 Hz, 2H, benzene H), 7.90 (d, *J* = 8.1 Hz, 2H, benzene H), 7.64–7.52 (m, 3H, benzene and pyridine H); ^13^C-NMR (DMSO-*d_6_*): δ 159.32, 147.12, 145.62, 138.80, 136.49, 132.76, 131.89, 130.51, 129.02, 124.07, 120.20, 117.48, 116.13, 112.32, 14.54; IR (KBr): *ν* 3282.8, 2918.3, 1654.9, 1544.9, 1490.9, 1388.7, 1224.8, 1080.1 cm^−1^; MS(ESI): *m/z* 347 [M+H]^+^; Anal. Calc. for C_17_H_13_F_3_N_4_O: C, 58.96; H, 3.78; N, 16.18. Found: C, 58.78; H, 3.55; N, 16.02.

### 3.8. Antifungal Bioassays

The fungicidal activity of the target compounds **6a**–**l** and **10a**–**k** were tested *in vitro* against *G. zeae*, *F. oxysporium*, and *C. mandshurica*, and their relative inhibitory ratio (%) was determined using the mycelium growth rate method [19,20]. Carboxin and boscalid were used as controls. After the mycelia grew completely, the diameters of the mycelia were measured, and the inhibition rate was calculated according to the formula:

*I* = (*D_1_* − *D_2_*)/*D_1_* × 100%

where *I* is the inhibition rate, *D_1_* is the average diameter of mycelia in the blank test, and *D_2_* is the average diameter of mycelia in the presence of those compounds. The inhibition ratios of those compounds at the dose 100 μg/mL have been determined, and the experimental results are summarized in Table 1.

Compound **6b** has been tested against three pathogenic fungi (*Gibberella zeae*, *F. oxysporum*, *C. mandshurica*,) at concentrations of 100, 50, 25, 12.5, 6.25, 0 μg/mL. The EC_50_ (effective dose for 50% inhibition μg/mL) values were estimated statistically by Probit analysis with the help of the Probit package of the SPSS software version 11.5 using a personal computer. The average EC_50_ was taken from at least three separate analyses for inhibition of growth using the basic EC_50_ program of SPSS version 11.5.

## 4. Conclusions

In conclusion, a novel series of pyrazolecarboxamide compounds containing an active 3-trifluoromethyl-1*H*-pyrazole-4-carboxamide skeleton were designed and synthesized using penthiopyrad as the lead structure. All target compounds were characterized by spectral data (^1^H-NMR, ^13^C-NMR, IR, MS) and elemental analysis. The compounds were tested *in vitro* for antifungal activity against three kinds of phytopathogenic fungi (*Gibberella zeae*, *Fusarium oxysporum*, *Cytospora mandshurica*). The results showed that some of the synthesized *N*-(substituted pyridinyl)-1-methyl-3-trifluoromethyl-1*H*-pyrazole-4-carboxamide compounds exhibited moderately antifungal activity, and compounds **6a**, **6b** and **6c** displayed more than 50% inhibition activities against *G. zeae* at 100 μg/mL, which were better than that of the commercial fungicides carboxin and boscalid. The preliminary structure-activity relationship analysis indicated that the antifungal activity of the synthesized compounds was obviously decreased when a methyl at the 1-position of the pyrazole ring was substituted by a phenyl. The structures of the target products need to be optimized to enhance their antifungal activity. Future structural modification and biological evaluation to explore the full potential of this novel class of antifungal molecules are under way.
